# A multicenter, randomized controlled, non-inferiority trial, comparing nasal continuous positive airway pressure with nasal intermittent positive pressure ventilation as primary support before minimally invasive surfactant administration for preterm infants with respiratory distress syndrome (the NIV-MISA-RDS trial): Study protocol

**DOI:** 10.3389/fped.2022.968462

**Published:** 2022-07-29

**Authors:** Hui Zhang, Jun Li, Lin Zeng, Yajuan Gao, Wanjun Zhao, Tongyan Han, Xiaomei Tong

**Affiliations:** ^1^Department of Pediatrics, Peking University Third Hospital, Beijing, China; ^2^School of Health Humanities, Peking University, Beijing, China; ^3^Research Center of Clinical Epidemiology, Peking University Third Hospital, Beijing, China; ^4^Department of Pediatrics, Beijing Hospital, Beijing, China

**Keywords:** nasal continuous positive airway pressure, nasal intermittent positive pressure ventilation, minimal invasive surfactant administration, neonatal respiratory distress syndrome, preterm infants

## Abstract

**Background:**

Non-invasive ventilation (NIV) treatment has been developed to minimize lung damage and to avoid invasive mechanical ventilation (IMV) in preterm infants, especially in those with a gestational age of <30 weeks. Our hypothesis is that for preterm infants <30 weeks with potential to develop respiratory distress syndrome (RDS), nasal continuous positive airway pressure (NCPAP) is non-inferior to the nasal intermittent positive pressure ventilation (NIPPV) as primary respiratory support before minimal invasive surfactant administration (MISA).

**Methods and design:**

The NIV-MISA-RDS trial is planned as an unblinded, multicenter, randomized, non-inferiority trial at 14 tertiary neonatal intensive care units (NICUs) in China. Eligible infants are preterm infants of 24–29^+6^ weeks of gestational age who have spontaneous breaths at birth and require primary NIV support for RDS. Infants are randomized 1:1 to treatment with either NCPAP or NIPPV once admitted into NICUs. If an infant presents progressively aggravated respiratory distress and is clinically diagnosed as having RDS, pulmonary surfactant will be supplemented by MISA in the first 2 h of life. The primary outcome is NIV treatment failure within 72 h after birth. With a specified non-inferiority margin of 10%, using a two-sided 95% CI and 80% power, the study requires 480 infants per group (in total 960 infants).

**Discussion:**

Current evidence shows that NIV and MISA may be the most effective strategy for minimizing IMV in preterm infants with RDS. However, there are few large randomized controlled trials to compare the effectiveness of NCPAP and NIPPV as the primary respiratory support after birth and before surfactant administration. We will conduct this trial to test the hypothesis that NCPAP is not inferior to NIPPV as the initial respiratory support in reducing the use of IMV in premature infants who have spontaneous breaths after birth and who do not require intubation in the first 2 h after birth. The study will provide clinical data for the selection of the initial non-invasive ventilation mode in preterm infants with a gestational age of <30 weeks with spontaneous breaths after birth.

**Clinical trial registration:**

https://register.clinicaltrials.gov, identifier: NCT05137340.

## Background

Although nearly 90% of newly born infants breathe spontaneously within 30–60 s after birth, the remaining 10% of newborns do not breathe within the first 60 s after birth or are persistently bradycardic (1). Because of intrauterine risk factors and the attack of preterm birth, very-low-birth-weight infants may have inhibited respiratory center, poor inspiratory effort, weak intercostal muscles, and poor diaphragmatic function (2). They may require positive pressure ventilation or even advanced resuscitation with an endotracheal tube. With aeration of the lungs, the liquid in the trachea and airways will be cleared and spontaneous breathing is triggered. Pulmonary surfactant (PS) is evenly distributed in the alveoli to reduce surface tension, reduce vascular resistance and improve pulmonary compliance (3). However, the alveolar type 2 cells in very preterm infants are immature, and the secretion of pulmonary surfactant is insufficient to meet the need, resulting in alveolar collapse and progressive exacerbation of dyspnea shortly after birth and in the first 2–6 h (4). Therefore, very preterm infants have the potential to develop respiratory distress syndrome (RDS).

According to the guidelines of neonatal resuscitation, positive end-expiratory pressure provides low positive pressure to the airway, which prevents lung collapse at the end of expiration and maintains functional residual capacity (2). For some very preterm infants, the birth transition process is smooth, and they can have spontaneous breaths after birth. Studies have shown that the immediate start (<15 min after birth) of nasal continuous positive airway pressure (NCPAP) may help to reduce the need for intubation and intermittent positive pressure ventilation (IPPV) in very preterm or very low birth weight infants and to prevent bronchopulmonary dysplasia (BPD) (5, 6). Therefore, it is proposed in the neonatal resuscitation guidelines in 2020 that for very preterm infants with spontaneous breaths after birth, NCPAP, a less invasive respiratory support, should be given, instead of intubation, to keep the lung open and delay the occurrence of lung collapse (2).

At present, NCPAP and nasal intermittent positive pressure ventilation (NIPPV) are the two most common non-invasive ventilation (NIV) modes for the initial respiratory support in NICUs (7). NIPPV imposes no more than 30 times per minute ventilator inflations to a set peak pressure (maximum 15 cmH_2_O) and may be delivered by short binasal prongs to very preterm infants. NIPPV has been used in NICUs for a variety of indications, especially for extubation ventilation support. It could reduce intubation due to frequent primary apnea, asynchronous thoracoabdominal motion, and inspiratory effort, and improve the stabilization of the chest wall and the tidal and minute volumes (8, 9). However, compared with NCPAP, NIPPV might be more invasive and might involve more risks to very preterm infants. An association has been found between the use of NIPPV and the increased risk of abdominal distention, vomiting and gastrointestinal perforation (10, 11). In view of the gentle ventilation support strategy to the tiny, very preterm infants during the birth transition period, there is lack of high-quality evidence to support the superiority of NIPPV to NCPAP.

RDS remains a significant problem for preterm infants, although its management has evolved in the “post-surfactant era”, resulting in improved survival of the very preterm infants (12). The optimal approach to administering pulmonary surfactant (PS) has attracted great attention in neonatology in recent years. The transition from intubation-surfactant-extubation (InSurE) to less-invasive surfactant administration (LISA) and minimally-invasive surfactant therapy (MIST), and the minimal invasive surfactant administration (MISA) with specially designed minimally invasive tubes in China, as well as administration of PS with laryngeal masks and aerosol inhalation, reflects the efforts to minimize lung trauma and the application of gentle ventilation strategy, as much as possible, in the care of very preterm infants in NICU (13, 14).

The updated *European Consensus Guidelines on the Management of Respiratory Distress Syndrome-*−*2019 Update* recommends NCPAP combined with LISA as the main treatment of respiratory distress syndrome (RDS) in preterm infants with spontaneous breaths (6). Unfortunately, there are few large randomized controlled trials to compare the effectiveness of NCPAP and NIPPV as the initial respiratory support mode before MISA in the treatment of preterm infants with RDS. A small-scale prospective cohort study suggests that the efficacy of LISA with NIPPV, in terms of the need of intubation, was not significantly different from that of NCPAP (15). A randomized controlled study shows that NIPPV combined with MIST reduced the need for invasive mechanical ventilation (IMV) within the first 72 h of life in infants with a gestational age of 26–32 weeks when it was compared with NCPAP although the reduction of the need for IMV was not found in the subgroup of infants aged <30 weeks (16). Hence, well-designed and larger randomized controlled studies are warranted for infants with a gestational age of <30 weeks.

The aim of this study is to assess whether NCPAP is non-inferior to NIPPV in preventing NIV treatment failure, when used as primary respiratory support for preterm infants <30 weeks and diagnosed with RDS.

## Methods

### Study design

**Figure 1:**
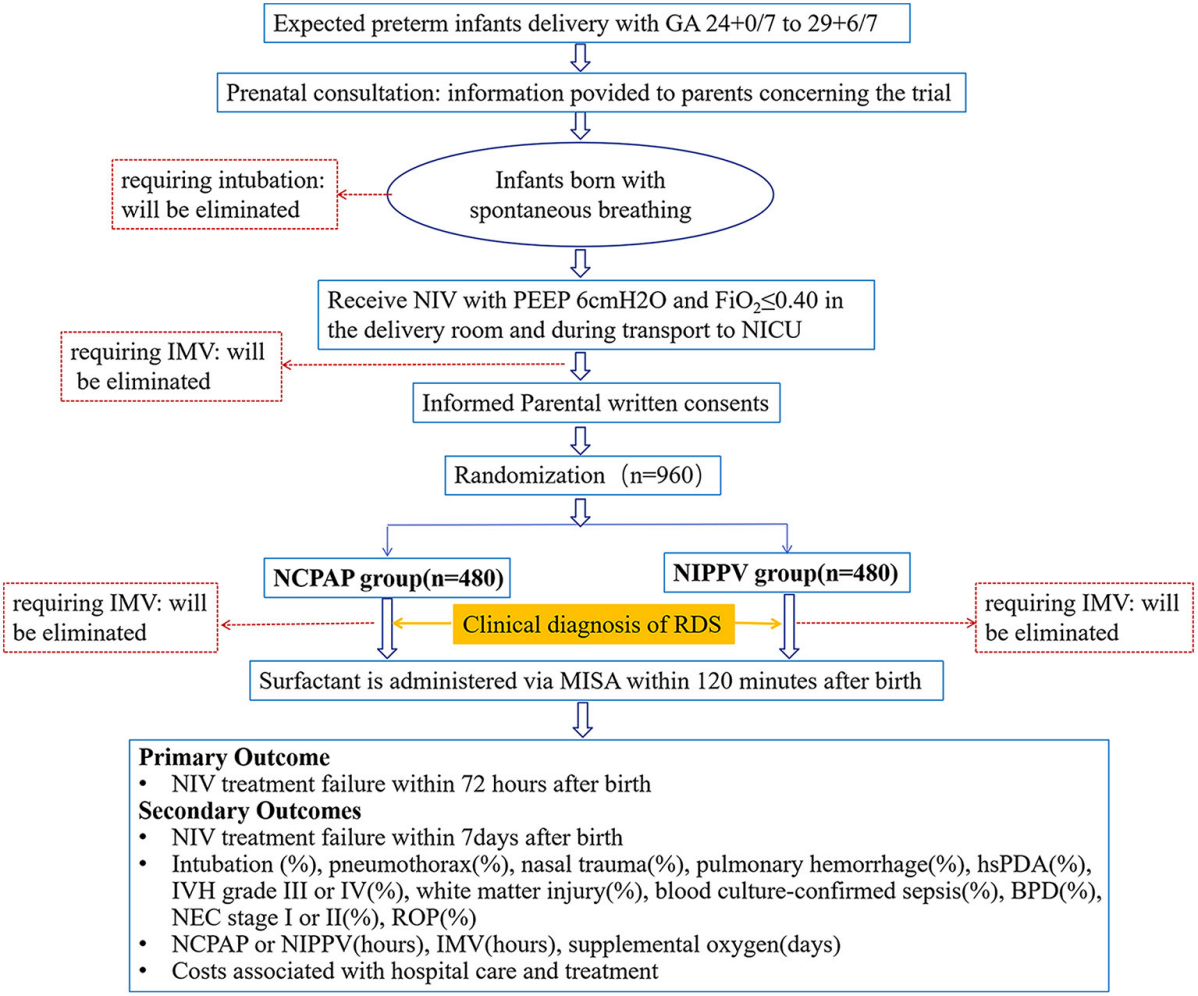
Prospective flow chart of the study. NCPAP, nasal continuous positive airway pressure; NIPPV, non-invasive positive pressure ventilation; GA, gestational age; IMV, invasive mechanical ventilation; NIV, non-invasive ventilation; RDS, respiratory distress syndrome; MISA, minimally invasive surfactant administration; hsPDA, hemodynamically significant patent ductus arteriosus; IVH, intraventricular hemorrhages; BPD, bronchopulmonary dysplasia; NEC, necrotizing enterocolitis; ROP, retinopathy of prematurity.

The NIV-MISA-RDS trial is a multicenter, randomized, non-inferiority trial, conducted in preterm infants <30 weeks' gestational age requiring primary non-invasive respiratory support for respiratory distress in the first 2 h of life. The trial will be conducted in 14 centers and carried out according to the prospective trial flow shown in Figure 1. All hospitals where the participating NICUs are located are midwifery institutions with an annual delivery of more than 3,000 newborns. All the participating NICUs are level three facilities with a more than 50% survival rate of premature infants 24–30 weeks in the past 3 years before the initiation of the study. Before delivery, a multidisciplinary consultation will be made, and a neonatologist will communicate with the family, as premature deliveries at 24–30 weeks involves many risks. A neonatal resuscitation team will be set up before the deliveries for the implementation of neonatal resuscitation with T-piece, non-invasive ventilation for preterm infants with spontaneous breaths. The neonatal resuscitation teams will use the transfer warmer for in-hospital transfer, and will use the T-piece device during transfer until admission of infants into NICUs. All infants will start either NCPAP or NIPPV by ventilators in NICUs after randomization. Pulmonary surfactant will be made available, and once diagnosis of RDS is made, PS will be administered within 120 min after birth. All neonatologists in the 14 centers have routinely taken care of preterm infants with respiratory distress and have used NIV as their standard mode of primary respiratory support. They have been trained for neonatal resuscitation and MISA. Individual practice and team training are required to sustain knowledge and skills.

### Participants

#### Inclusion criteria

Infants who meet all of the following criteria will be included:

1. Infants of 24–29^+6^ weeks GA.
2. Infants with spontaneous breaths and signs of respiratory distress that will receive non-invasive respiratory support [positive end expiratory pressure (PEEP) of 6 cmH_2_O and fraction of inspired oxygen (FiO_2_) ≤ 0.40] immediately after birth in a delivery room and during transfer to a NICU.
3. They have a clinical diagnosis of RDS within 2 h of life. The diagnosis of RDS will be based on clinical manifestations (respiratory rate > 60/min, nasal flaring, retractions, grunting, or skin cyanosis) and (FiO_2_) > 0.3 for (SpO_2_) > 85%, or the Silverman Anderson Score (SAS) > 5 points, or the SAS increases > 2 points per hour.
4. Parental written consent is obtained.

#### Exclusion criteria

Infants who meet any of the following criteria will be excluded:

1. Infants who have been intubated prior to pulmonary surfactant administration due to postnatal resuscitation or other reasons.
2. Infants with obvious malformations affecting respiratory function.
3. Infants who have been transferred to other hospitals for surgery or die of other complications with uncompleted data.
4. Infants who have participated in other interventional research.

### Randomization

After transfer to NICU wards, eligible infants will be randomly assigned in a 1:1 ratio to receive either NCPAP (experimental group) or NIPPV (active control group) as the initial respiratory support. Treatment allocations will be based on a pre-specified random number table and concealed in sealed opaque envelopes. The treatment allocations sequence will be generated using computer programs (R software) by an independent statistician. Multiple-birth infants will be randomized separately.

### Quality control

This trial follows the Good Clinical Practice (GCP) standards. This trail also meets the requirements of the Ethics Committees and is carried out under the supervision of the Ethics Committees of the participating institutions. A Data Safety Monitoring Board (DSMC) will be established, consisting of two independent neonatologists and an independent statistician. DSMC review points will be set up for trial progress and safety.

### Blinding

Operators, clinicians and nurses will not be blinded to the intervention allocation, but independent outcome evaluators and data analysts will be blinded to the allocation. To reduce bias, predefined objective criteria will be specified for the main outcomes of treatment failure in order to provide clear guidance to outcome evaluators.

### Assessment points

The assessments will be performed at predetermined points after randomization (Table 1). The primary end point is at 72 h after birth. Changes in Silverman Anderson Score, oxygen saturation, blood gas, respiratory support mode, ventilator parameters and/or outcomes will be monitored and documented at allocation, at pre-surfactant administration, and at 12 h, 24 h, 48 h, 72 h, 7 days after allocation, at a post-menstrual age of 36 weeks, and at discharge.

**Table 1:** List of measures and assessment points.

**Measure**	**Assessment point**								
	**Allocation**	**Post-allocation**							
	**Within 0.5 h** **after birth**	**Pre-surfactant** **administra-tion**	**12 h**	**24 h**	**48 h**	**72 h**	**Day 7**	**PMA 36**	**Discharge**
Clinical index									
Silverman Anderson score	×	×	×	×	×	×	×		
Oxygen saturation	×	×	×	×	×	×	×	×	×
Blood gas		×		×	×	×			
Respiratory support mode	×	×	×	×	×	×	×	×	×
Ventilator parameters	×	×	×	×	×	×	×	×	×
Outcomes assessment									
NIV treatment failure						×	×		
intubation (%)			×	×	×	×	×	×	×
Pneumothorax (%)			×	×	×	×	×	×	×
Nasal trauma (%)			×	×	×	×	×	×	×
Pulmonary hemorrhage (%)			×	×	×	×	×	×	×
hsPDA (%)			×	×	×	×	×	×	×
IVH grade III or IV (%)			×	×	×	×	×	×	×
White matter injury (%)			×	×	×	×	×	×	×
Blood culture-confirmed sepsis (%)			×	×	×	×	×	×	×
BPD (%)								×	
NEC stage I or II (%)			×	×	×	×	×	×	×
ROP (%)								×	×
NCPAP or NIPPV (hours)									×
IMV (hours)									×
Supplemental oxygen (days)									×
Costs associated with hospital care									×

*NIV, non-invasive ventilation; hsPDA, hemodynamically significant patent ductus arteriosus; IVH, intraventricular hemorrhages; BPD, bronchopulmonary dysplasia; NEC, necrotizing enterocolitis; ROP, retinopathy of prematurity; NCPAP, nasal continuous positive airway pressure; NIPPV, non-invasive positive pressure ventilation; IMV, invasive mechanical ventilation*.

### Planned intervention

Preterm infants with spontaneous breaths will be stabilized on non-invasive respiratory support (PEEP of 6 cmH_2_O and FiO_2_ ≤ 0.40) in the delivery room and during admission to NICUs, and then randomly selected to start NCPAP or NIPPV within 30 min of birth.

#### NCPAP group

The ventilators parameter of the NCPAP group will be set with a PEEP of 6 cmH_2_O (adjustment range 6–8 cmH_2_O) and an FiO_2_ of 0.21–0.40, in order to maintain an oxygen saturation level of 90–95%.

#### NIPPV group

NIPPV will be set in a synchronized mode with a PEEP of 6 cmH_2_O (adjustment range 6–8 cmH_2_O), peak inspiratory pressure (PIP) of 15 cmH_2_O (regulation range 15–20 cmH_2_O), inspiratory time of 0.3 s (regulation range 0.3–0.4 s), respiratory rate of 30 times/min (regulation range 20–40 times/min) and FiO_2_ of 0.21–0.40.

#### MISA method

Under NCPAP or NIPPV, the calf pulmonary surfactant (Calf pulmonary surfactant, Beijing Double-crane pharmaceutical Co. LTD) will be administered *via* MISA method within 120 min after birth if infants are clinically diagnosed with RDS. While the infants are on ventilator support by NCPAP or NIPPV, a direct laryngoscope and a specifically designed thin catheter with 1.67 mm in diameter (LISA catheter^®^, Beijing Double-crane pharmaceutical Co. LTD) will be used to finish the surfactant administration. A direct laryngoscope will be introduced to provide a glottal view before the operator inserts the LISA catheter^®^ 0.5–1.0 cm below the inverted V-shaped vocal cords. After withdrawing the laryngoscope, the operator will fix the thin catheter at the infant's mouth corner with the forefinger and thumb, and the end of the LISA catheter^®^ will be connected to a 5 ml syringe containing surfactant solution. The surfactant will be administered through the LISA catheter^®^ by a nurse within 120–300 s by mini-boluses. The LISA catheter^®^ will be immediately removed after surfactant administration. If an infant develops bradycardia or desaturation (SPO_2_ <85%), stop surfactant administration, keep the LISA catheter^®^ in place, gently touch the infant's back to stimulate spontaneous breaths, increase FiO_2_ and wait for the heart rate and SPO_2_ to return to normal range before finishing the remaining surfactant with cautions. If the heart rate and SPO_2_ continue to drop with the occurrence of apnea, the NIPPV or NCPAP ventilator should be used for positive pressure ventilation immediately, and the operation will be continued after heart rate and SPO_2_ return to normal. If surfactant regurgitation occurs, suspend administration and slow down the infusion until there is no regurgitation in the mouth.

#### Therapy

The first dose of pulmonary surfactant is 100 mg/kg, and the criteria of repeated use of surfactant are as follows: (1) within 72 h after birth; (2) progressively aggravated respiratory distress after first dose with a minimum interval of 4–6 h; (3) chest X-ray is consistent with the imaging findings of RDS; (4) dyspnea caused by other causes is excepted.

The decision for intubation and IMV within 72 h and 7 days after birth of life is based on the following criteria: (1) severe respiratory acidosis with PaCO_2_ > 65 mmHg and pH <7.2; (2) hypoxemia with SpO_2_ of <0.85 on FiO_2_ > 0.4; (3) recurrent apnea with ≥3 episodes per hour or a single episode of apnea requiring positive pressure ventilation within 24 h; (4) SAS increasing > 2 points per hour, or SAS > 5 points lasting more than 2 h after first or second dose of surfactant administration; (5) pulmonary hemorrhage; (6) cardiopulmonary arrest requiring intubation; (7) neonatologists in the NICU believe that invasive respiratory support should be given to patient. Extubation criteria are established as mean airway pressure (MAP) <8 cm H_2_O and FiO_2_ <0.3.

The decision for weaning of NCPAP or NIPPV is based on the following criteria: (1) the infant has recovered from RDS; (2) the signs of respiratory distress have disappeared and SAS <3 points; (3) when NCPAP with PEEP <3–5 cmH_2_O, or when NIPPV with MAP <6–7 cmH_2_O and FiO_2_ ≤ 25%; (4) without apnea or intermittent hypoxia.

After being assessed by the NICU clinicians to meet all the conditions and lasting for more than 24 h, withdrawal of NIV can be considered.

All of the enrolled infants will be started on caffeine soon after admission at a loading dose of 20 mg/kg followed by a maintenance dose of 5–10 mg/kg.

### Outcome measures

#### Primary outcome measure

The primary outcome is NIV treatment failure within 72 h after birth, as determined by objective oxygenation, blood gas, and apnea criteria, or the need for intubation and mechanical ventilation.

#### Secondary outcome measures

The secondary outcomes are as follows:

1. NIV treatment failure within 7 days after birth
2. Rate of intubation
3. Rate of pneumothorax
4. Rate of nasal trauma
5. Rate of pulmonary hemorrhage
6. Rate of hemodynamically significant patent ductus arteriosus (hsPDA)
7. Rate of intraventricular hemorrhages (IVH, grade III or IV)
8. Rate of white matter injury
9. Rate of blood culture-confirmed sepsis
10. Rate of bronchopulmonary dysplasia (BPD)
11. Rate of necrotizing enterocolitis (NEC, ≥stage II)
12. Rate of retinopathy of prematurity (ROP)
13. Duration of NCPAP/NIPPV, duration of IMV, and days on supplemental oxygen
14. Costs associated with hospital care and treatment.

#### Data collection and diagnoses

Patient demographic data mainly include: gestational age, birth weight, sex, multiple births, antenatal corticosteroids use, mode of delivery, maternal complications, Apgar score and cord blood gas. Clinical data mainly include: SAS, requiring more than one dose of surfactant, blood gas, the need for IMV within 72 h and 7 days after birth, duration of NCPAP/NIPPV, days on IMV, days on supplemental oxygen, costs associated with hospital care, morbidity of BPD, pneumothorax, nasal trauma, pulmonary hemorrhage, hsPDA, IVH, white matter injury, blood culture-confirmed sepsis, NEC and ROP.

The clinical diagnosis of RDS will be based on clinical manifestations (respiratory rate > 60/min, nasal flaring, retractions, grunting, or skin cyanosis) and (FiO_2_) > 0.3 for (SpO_2_) > 85%, or SAS > 5 points or SAS increasing > 2 points per hour (17). BPD will be diagnosed according to the National Institutes of Health criteria (18). Pneumothorax will be diagnosed by the presence of air in the pleural cavity in a chest X-ray. Pulmonary hemorrhage will be diagnosed based on the gushing of bloody fluid from the upper airway or endotracheal intubation and when the chest X-ray is consistent with the relevant clinical findings. The diagnosis for hsPDA will be based on clinical signs and echocardiogram (19). IVH and white matter injury will be diagnosed by cranial ultrasound examination, and IVH will be graded by the Papile classification system (20). Blood culture-confirmed sepsis will be defined as clinical sepsis with evidence of pathogens in blood culture. NEC will be graded according to the modified Bell's classification system (21). The diagnosis and staging of ROP will be based on retinal examination by a consultant ophthalmologist.

### Safety procedures

All eligible infants will be monitored by researchers throughout the study to ensure their safety. Any serious adverse events or important adverse events will be assessed by the neonatologists in each center and by the principal investigator. This study adheres to the principle of subject protection and discontinues the experiment in time if any adverse events occur, regardless of whether they are related to this study or not.

### Sample size calculation

The failure rate of NCPAP with MISA approach was 24.5% (of NIPPV, 22.4%) in preterm infants aged <32 weeks with RDS (15). There was no statistical difference between the two groups. We have set the non-inferiority margin for the trial at 10%. That is, NCPAP is supposed to be non-inferior to NIPPV if the difference in risk of NCPAP treatment failure and the upper limit of its bilateral 95%CI is <10%. To demonstrate this with 80% power, 452 infants per study group are required. In order to prevent the loss of samples, the sample size is increased by 6%. Therefore, a total of 960 samples will be selected and allocated randomly 1:1 into the two study groups (480 in the NCPAP group and 480 in the NIPPV group).

### Statistical analysis

#### Baseline data

Demographic and clinical characteristics for the NCPAP group and the NIPPV group at baseline will be described in mean and standard deviation, median and range, or rate and percentage. Continuous variables will be presented in mean ± standard deviation or median (minimum-maximum), and categorical variables will be presented in numbers and proportions. We will not test the hypothesis with the two sets of baseline data.

#### Outcome data

The data will be analyzed based on the original treatment allocation of the participants in accordance with the intention-to-treat principle. To compare the primary and secondary outcomes between the two groups, we will use the Student's *t*-test for continuous variables (if normally distributed), the Mann-Whitney U test (if skewed) and the *chi*-squared test for categorical variables. In addition to the *p*-value of the outcomes, results are to be given in terms of differences and 95% CIs. Preplanned exploratory subgroup analyses are to be performed for the primary and other pre-specified outcomes by gestational age, and birth weight. All data will be analyzed using SPSS version 20.0. Two-tailed *p-*values <0.05 will be considered as statistically significant.

## Discussion

In recent years, most studies have shown that NIV reduces the risk of BPD in preterm infants when compared to intubation and IMV (5, 22). Previous trials have reported that MISA during NIV further minimized the need for IMV and the incidence of BPD in very low birth weight infants with RDS (23, 24). NIV modes commonly used in NICUs include NCPAP, NIPPV, bi-level positive pressure and high flow nasal cannula, of which NCPAP and NIPPV are the two most common modes (7, 25). The goal of NCPAP is to maintain the functional residual capacity for reducing breathing effort. Early use of NCPAP is an alternative to intermittent positive-pressure ventilation and intubation (26). However, NCPAP has a high failure rate and requires IMV in the first 72 h−7 days of life, especially in extremely low birth weight infants. This is due to the immature respiratory center, poor diaphragmatic strength and chest wall collapse in these infants (27). NIPPV superimposes an intermittent peak pressure on NCPAP. Some studies have shown that NIPPV is superior to NCPAP after extubation (8, 28, 29). A Cochrane meta-analysis of 10 studies involving 1,431 infants compared the effects of NIPPV and NCPAP on respiratory support after extubation and reported that NIPPV reduced the incidence of respiratory respiratory support failure [RR: 0.70 (95% CI 0.60, 0.80)] and reintubation [RR: 0.76 (95% CI 0.65, 0.88)] more effectively than NCPAP (8). Ferguson et al. suggested that NIPPV was more effective than NCPAP in preventing extubation failure (30).

At present, it is still controversial whether NIPPV is superior to NCPAP in initial non-invasive respiratory support for premature infants, especially for those with a gestational age of <30 weeks. A meta-analysis of 1,061 infants recruited in 10 trials shows that early NIPPV seems to reduce the need for intubation and IMV in premature infants with RDS than NCPAP alone (9). A small clinical study shows that initial NIPPV enhances the beneficial effects of NCPAP as non-invasive respiratory support, contributing to reducing the risk of treatment failure in very low birth weight infants with RDS (31). However, one large randomized controlled trial involving 1,009 patients with a birth weight of <1,000 g, comparing NIPPV with NCPAP, shows no difference in non-invasive support failure (58.3 vs. 59.1%) and survival with BPD (33.9 vs. 31.0%) (32). In a multicenter retrospective cohort study involving 512 preterm infants with RDS, NIPPV was not found to be more effective than NCPAP in decreasing the need for IMV within the first 7 days of birth (33). A randomized controlled trial shows that among infants born between 26 and 32 weeks of gestation, NIPPV as the initial respiratory support with MIST reduced the need for IMV (13 vs. 29%; *p* = 0.005) and the rate of moderate-to-severe BPD (7 vs. 16%; *p* = 0.046) compared with NCPAP, however, this was not seen for the subgroup of preterm infants of a gestational age of <30 weeks (20 vs. 32%; *p* = 0.16) (16). Therefore, carefully designed and larger randomized controlled trials are needed for infants of a gestational age of <30 weeks.

In this multicenter randomized controlled trial, we put forward the hypothesis that NCPAP is not inferior to NIPPV as the initial respiratory support before MISA in reducing the use of IMV in preterm infants aged <30 weeks. The study will provide some data for the selection of initial NIV mode in preterm infants with a gestation age of <30 weeks with spontaneous breaths. Nevertheless, there are some potential limitations in our study design: (1) Preterm infants with spontaneous breaths will not be grouped immediately after birth, but upon arrival in NICUs. We stipulate that non-invasive respiratory should be supported with transport ventilator or T-piece during delivery room resuscitation and transfer, and we will limit the time to arrive at the NICUs to within 30 min after birth. (2) Treatment allocations (in a 1:1 ratio) will be based on a pre-specified random number table, without stratification according to gestational age and birth weight. This is due to the fact that the estimated sample size of premature infants at 24–26 weeks with spontaneous breaths after birth and without intubation during delivery room resuscitation is relatively small. (3) The diagnosis of RDS will be made based on clinical manifestations and Silverman Anderson Score, without classification of RDS severity.

## Conclusion

If, as expected, NCPAP is non-inferior to NIPPV, it will provide an opportunity to increase the availability of NCPAP treatment for preterm infants of a gestational age of <30 weeks with spontaneous breaths.

## Ethics statement

The studies involving human participants were reviewed and approved by Peking University Third Hospital Medical Science Research Ethics Committee (Project Number: M2021378). Written informed consent to participate in this study was provided by the participants' legal guardian/next of kin.

## Author contributions

HZ and TH: concept and design. HZ, TH, LZ, YG, and WZ: acquisition, analysis, and interpretation of data. HZ, TH, and JL: drafting of the manuscript. HZ and LZ: statistical analysis. TH and XT: supervision. All authors: critical revision of important intellectual content in the manuscript. All authors contributed to the article and approved the submitted version.

## Conflict of interest

The authors declare that the research was conducted in the absence of any commercial or financial relationships that could be construed as a potential conflict of interest.

## Publisher's note

All claims expressed in this article are solely those of the authors and do not necessarily represent those of their affiliated organizations, or those of the publisher, the editors and the reviewers. Any product that may be evaluated in this article, or claim that may be made by its manufacturer, is not guaranteed or endorsed by the publisher.
